# Environmentally Friendly Fertilizers Based on Starch Superabsorbents

**DOI:** 10.3390/ma12213493

**Published:** 2019-10-25

**Authors:** Orietta León, Diana Soto, Jesús González, Carlos Piña, Alexandra Muñoz-Bonilla, Marta Fernandez-García

**Affiliations:** 1 Laboratorio de Polímeros y Reacciones, Escuela de Ingeniería Química, Facultad de Ingeniería, Universidad del Zulia, Sector Grano de Oro, 4011 Maracaibo, Venezuela; dsoto@fing.luz.edu.ve (D.S.); gonzalezsj4@gmail.com (J.G.); 30carlospina@gmail.com (C.P.); 2 Departamento de Química y Propiedades de Materiales Poliméricos, Instituto de Ciencia y Tecnología de Polímeros (ICTP-CSIC), C/ Juan de la Cierva 3, 28006 Madrid, Spain; sbonilla@ictp.csic.es; 3 Interdisciplinary Platform for Sustainable Plastics towards a Circular Economy-Spanish National Research Council (SusPlast-CSIC), 28006 Madrid, Spain

**Keywords:** starch, graft copolymers, adsorption, superabsorbent, controlled release

## Abstract

Superabsorbents starches (SASs) were synthesized and characterized starting from native corn starch, bitter cassava and sweet cassava by graft copolymerization with itaconic acid. Additionally, their swelling behavior was studied both in water and in buffer solutions with different pHs and saline solutions. Their applicability was tested as environmentally friendly fertilizers in the absorption and release of urea, potassium nitrate and ammonium nitrate at different concentrations of fertilizers. The values of swelling at the equilibrium (H^∞^) in water and different media of the graft copolymers demonstrated their superabsorbent capacity, polyelectrolyte behavior, and smart response to environmental stimuli. The percentage of fertilizer absorbed and released from the SASs was a function of the initial concentration of the fertilizer in the medium. The loading and release of SASs were depended on the initial concentration of the fertilizer in the medium as well as the nature, structure, and morphology of the starch used.

## 1. Introduction

New technologies developments in agriculture have generated greater fertilizer and water inputs. Production per unit of land has greatly increased what has allowed increasing economic developments. However, while these developments have been significant, the environmental impact has been excessive. Such costs associated with agricultural development have resulted from excessive application of fertilizers, which lead to eutrophication and water toxicity, pollution of irrigation water, air pollution (NH_3_, among others), degradation of quality of soil and changes in the ecosystem, resulting in the hydrolysis of nutrients and the action of microorganisms, thus questioning the sustainability of modern agriculture [1,2]. Therefore, new ecofriendly treatments are necessary to increase production without compromising the environment. This can be achieved by efficiently increasing the use of fertilizers (nitrogen, phosphate, and potassium) and water as well as the integrated use of crop system management.

There are a variety of strategies that are used to increase the efficient use of fertilizers and eliminate their negative impact on the environment. These improved fertilizer application methods include the use of the slow or localized application, precision fertilization, irrigation systems via fertirrigation-fertilization and the use of environmentally friendly fertilizers [3,4]. 

Environmentally friendly fertilizers (EFFs) offer an effective way to improve nutrient efficiency, minimize losses by leaching and volatilization of fertilizers, and reduce environmental hazards. They reduce environmental pollution due to nutrient losses through the retardation or controlled release of nutrients into the soil. Normally, EFFs are formulated so that the nutrient is coated with environmentally friendly materials, which can be degraded in the soil and converted into carbon dioxide, water, methane, inorganic compounds or microbial biomass. While it is true that this is the most common and commercially available formulation, other technologies have been used to develop EFFs, such as the use of SASs where the nutrient is entrapped or composites prepared by blending polymers with fertilizers, among others.

The superabsorbent polymers based on biopolymers, such as chitosan, cellulose, and starch, among others, in addition to being environmentally friendly, they can biodegrade in the soil. SASs are hydrophilic polymer networks that can absorb and retain a large amount of water. They are potential materials to regulate fertilizer release behavior due to their electrical charge and interconnection of open channels in the network. These biopolymers have been confirmed as soil conditioning agents, which reduce the frequency of irrigation, erosion, and evaporation of water in hot and dry climates, also improve soil quality by lowering soil degradation [5,6,7,8]. 

Graft copolymerization, often using acrylic acid and acrylamide comonomers, converts starch into a SASs [9]. These comonomers, however, decrease the biodegradability of the material. Starch is a mixture of polysaccharides consisting of 20–30% amylose, 70–80% amylopectin, and a minor fraction (1 to 2%) of non-glucoside conformation. The most important conventional sources for starch extraction are cereal grains (corn, wheat, rice, and sorghum) and tubers (potato, yucca, sweet potato and sago), being also in leaves, seeds of legumes and fruits. Depending on the source of production, starch has several physicochemical, structural and functional characteristics that allow it to expand its range of uses in the industry. The most important root and tuber crops worldwide for obtaining starch are cassava, sweet potato, potato, yam, occumo [10]. 

This work presents the preparation of superabsorbent starches from different botanical sources of starch (corn, sweet yucca and bitter yucca) by grafting itaconic acid (IA) using the KMnO_4_/NaHSO_3_ redox system, which leads to the formation of carbonyl and carboxyl groups with minimum polymer hydrolysis and without reduction of polymer viscosity. IA is a diprotic acid of natural origin, which incorporated acid functional groups to the structure of starch capable of responding to external stimuli and interacting with chemical species in the environment [9]. These SASs were used for the first time as environmentally friendly nitrogen fertilizers, including urea, KNO_3_, and NH_4_NO_3_. Urea is the most worldwide popular and economical nitrogen fertilizer used. Compared to other sources, it contains 46% nitrogen, the highest concentration of nitrogen available. However, other types of nitrogen sources are also of great interest in agriculture. KNO_3_ is an excellent fertilizer that contains two primary nutrients [2]. 

## 2. Materials and Methods 

### 2.1. Materials

Native corn starch (NCS) provided by Alfonso Rivas and Cía (Caracas, Venezuela). Hydrochloric acid (HCl, 37%) from Merck (Darmstadt, Alemania), hydroxylamine hydrochloride (NH_2_OH HCl, 99%) from Loba Chemie (Mumbai, India), ethyl alcohol (CH_3_CH_2_OH, 95%), sodium hydroxide (NaOH, 99%), itaconic acid (C_3_H_4_(COOH)_2_), ≤99%), potassium nitrate (KNO_3_, ≥99%) from Merck (Darmstadt, Alemania), sodium chloride (NaCl, 99.8%) from Baker (Madrid, Spain), phosphoric acid (H_3_PO_4_, 85.5%) from Fisher Scientific (Madrid, Spain), ammonium chloride (NH_4_Cl, ≥99.5%), magnesium chloride (MgCl_2_, ≥98%), iron (III) chloride (FeCl_3_, 97%), potassium chloride (KCl, ≥99%), trisodium phosphate sodium dodecahydrate (Na_3_PO_4_ 12H_2_O, ≥98%), monobasic sodium phosphate anhydrous (NaH_2_PO_4_, 98–100.5%), ammonium nitrate (NH_4_NO_3_, ≥98%), urea (CH_4_N_2_O, 98%), from Sigma-Aldrich (St. Louis, Kansas, MO, USA), disodium phosphate anhydrous (Na_2_HPO_4_, 98–100.5%) from Scharlau (Barcelona, Spain), *p*-dimethylaminobelzaldehyde (C_9_H_11_NO, 99%) from Himedia (Mumbai, India), potassium permanganate (KMnO_4_, 99%), sodium bisulfite (NaHSO_3_, 66.9%) from Mallinckrodt (Staines, Reino Unido). All reagents were used as received.

### 2.2. Starches Isolation and Characterization

#### 2.2.1. Starches Isolation

The starches were extracted from cassava samples following methods recommended by Food and Agriculture Organization (FAO) of Unites Nations with slight modifications [11]. Starch was extracted from sweet cassava (NMUS) following a humid method. Initially, the cassava was washed, peeled and cut into small pieces (1 × 1 cm). The sample was then mixed with distilled water in a cassava/water ratio of 1/4. The mixture was milled for 2 min and subsequently was filtered through an 80 μm mesh sieve to remove the fibers and kept during 20 h at 25 °C. The supernatant was discarded, and the slurry was repeatedly washed with distilled water. Then, the slurry was centrifuged at 2500 rpm for 12 min and subsequently was dried at 40 °C in a hot air oven for 24 h. After that, the sample was grinded and sieved (100 μm mesh). The starch from bitter cassava (NMES) was isolated following a dry extraction method. Firstly, the cassava was washed, peeled, grated into small pieces. Later, the sample was placed on an 80 μm mesh sieve, washed with distilled water and pressed to obtain the slurry. The wash-press cycle was repeated until the water was transparent and the slurry was kept during 20 h at 25 °C. Then, it was followed the same procedure described for the sweet cassava. The yield of extraction in both cases was determined by weighing the starch obtained after drying. Extraction yield of NMUS = 15.8% and NMES = 8.2%.

#### 2.2.2. Amylose Content 

The amylose contents of cassava were analyzed following a standard method (ISO 6647-1:2015) [12] based on the appearance of blue color by the addition of an iodine reagent to starch under standardized conditions. 100 mg of sample was taken and gelatinized with shaking with 9 mL of 1 M NaOH in a heating bath at 95 °C for 15 min, allowed to cool to room temperature and transferred to a 100 mL round-flask to complete with water, it was stirred vigorously and this was called as dispersion A. Then, 2 mL of dispersion A was mixed in three 100 mL round-flask with 3 mL of 0.09 M NaOH and 6 mL of Lugol, completed with water, mixed vigorously, allowed to stand for 10 min and was named as dispersion B. Then, 5 mL of dispersion B was taken and the absorbance was measured using a Perkin Elmer UV-vis Lambda 2 (Perkin Elmer Inc., Wellesley, MA, USA) at 590 nm. The value of absorbance was entered into the calibration curve previously built for standard amylose [9]. Finally, the amylose content in the starch was determined by Equation (1):

(1) $$\mathrm{Amylose}\;\mathrm{content}\;(\%)\;=\;(\mathrm{Amylose}\;\mathrm{in}\;\mathrm{solution}\;(\%)/{\mathrm{mS})\;\times \;F}_{d}$$

where (amylose in solution (%)) is obtained in the absorbance measurement, mS is the weight of starch (g) and F_d_ is the dilution factor. The amylose content of NCS is 29 ± 1%, NMES is 30 ± 2% and NMUS is 27 ± 1%.

#### 2.2.3. Differential Scanning Calorimetry (DSC)

To obtain the thermometric parameters of the native starch, 10 mg of the corresponding sample and 7 μL of water (humidity percentage of 75%) were added in a high-pressure pan, sealed and allowed to stand for 24 h to promote the equilibrium. Likewise, a reference pan without sample or water was sealed and stored for the same period. After this time, the wet sample was heated from room temperature to 100 °C, at a heating rate of 5 °C/min in N_2_ flow in the Perkin Elmer DSC equipment (Pyris 6, Perkin Elmer Inc., Wellesley, MA, USA). Temperatures of gelatinization (T_p_) of NCS, NMES and NMUS are 71 °C, 78 °C, and 75 °C, respectively.

### 2.3. Preparation of Grafted Starches

The native starch was gelatinized into a bath at 80 °C by adding 10 g of sample in a three-neck flask together with 100 mL of water for 15 min with shaking and using a condensation system at 25 °C. Then, 10 mL of water was added, and it was allowed to cool down to 60 °C. To the gelatinized starch paste was added a 200 mL solution of 0.6385 g of KMnO_4_ and 1.5550 g of NaHSO_4_, starting the pre-oxidation of the substrate. After 10 min 12.12 g of AI were added and the volume of 500 mL was completed, initiating the copolymerization reaction, which proceeded with vigorous stirring for 3 h at 60 °C [13]. At the end of this time, the product was removed from the bath and allowed to cool down to room temperature. The purification of the product was done by precipitation in 500 mL of ethanol, then, the precipitate filtered and washed with a mixture of ethanol/water (50/50 v/v). Consecutive washings were carried out until negative tests were obtained for Baeyer and Lugol tests, as well as pH close to that of the washed solution. Finally, the samples were dried at 40 °C until constant weight.

### 2.4. Characterization of Materials

#### 2.4.1. Percentage of Insoluble Fractions, I (%)

For the determination of insoluble fractions, 100 mg of sample were initially weighed and immersed in 100 mL of distilled water without agitation at room temperature for 24 h. After that, the samples were filtered to extract the soluble fraction and dried at 40 °C until a constant weight was obtained. The relation between the final and initial mass of the sample allowed obtaining mathematically the insoluble fractions, I (%), according to Equation (2).

(2) $$I(\%)=\frac{W_{f}}{W_{0}}\times 100$$

where W_f_ and W_0_ correspond to the final and initial mass of the sample in mg, respectively.

#### 2.4.2. Content of Aldehyde and Carboxyl Groups

The content of groups –C(O)H and –COOH of the graft copolymers were determined volumetrically following the methodology described elsewhere [13]. The percentage of groups –C(O)H was obtained through Equation (3) and the percentage of –COOH groups by Equation (4).

(3) $$-C\left(O\right)H\;\left(\%\right)=\frac{{(V}_{B}-V_{M}{)\times C}_{\mathrm{HCl}}\times 0.028\times 100}{W_{D}}$$

(4) $$-\mathrm{COOH}(\%)=\frac{{(V}_{M}-V_{B}{)\times C}_{\mathrm{NaOH}}\times 0.045\times 100}{W_{D}}$$

where V_B_ and V_M_ are the titrant volumes in mL consumed by the blank and the starch sample in the respective assay, C_HCl_ is the concentration of HCl in mol/L, C_NaOH_ is the concentration of NaOH in mol/L and W_D_ is the mass of the dry sample in g. 

#### 2.4.3. Determination of Apparent Viscosity

The apparent viscosity of the starches was made over a shear rate from 50 to 1300 s^−1^ at 80 °C using a Haake rotovisco RV 20 (Thermo Fisher Scientific Inc., Waltham, MA, USA). 

#### 2.4.4. Nitrate Content in Water

The nitrate content [14] in water was carried out by spectrophotometry using a Perkin Elmer UV/VIS spectrometer Lambda 2 (Perkin Elmer Inc., Wellesley, MA, USA), at λ = 220 nm and 275 nm. Lambert Beer law is applicable up to a concentration of ca. 30–40 mg/L of NO_3_^−^ at λ_max_ = 220 nm. Sample and standard solutions were added 0.5 mL HCl 1 M before measured using a UV-Vis spectrophotometer. The concentration of the calibration standard solution was made from 0.03 to 5.0 mg/L. The calibration curve was made from the difference between absorbance data at 220 nm and multiple of absorbance data at 275 nm.

#### 2.4.5. Fourier Transform Infrared Spectroscopy (FTIR)

The infrared spectra of starches were recorded using a Fourier transform infrared spectrometer Shimadzu IR Prestige-21 (Shimadzu Corporation, Tokyo, Japan), in the region of 4000–400 cm^−1^. The spectra resolution was 2 cm^−1^ and 100 scans were averaged for each spectrum. Pellets were obtained by mixing the sample previously dried, with KBr at a ratio of 1:50 (sample: KBr). 

FTIR spectra of grafted starches, (KBr, cm^−1^): 3310–3298 cm^−1^ (νO–H, inter- e intramolecular interactions), 2930–2924 cm^−1^ (ν_a_C–H of the anhydroglucose units (AGU)), 2901–2885 cm^−1^ (ν_s_C–H of AGU ring), 2800 cm^−1^ (ν_s_C‒H of substituent), 1642–1633 cm^−1^ (δ_ip_ O–H of H_2_O strongly bonded), 1417–1407 cm^−1^ (δ_a_C–H of AGU), 1372–1367 cm^−1^ (δ_s_ C–H of AGU ring), 1336–1335 cm^−1^ (δ_ip_ O–H of C–OH), 1242–1238 cm^−1^ (νCH_2_–OH), 1050–1047 cm^−1^ (ν_a_ C–O–C of AGU ring), 1077–1076 cm^−1^ (δ_oop_ C–H), 1015–1012 cm^−1^ (ν_a_ C–O and δ_ip_ C–OH of primary hydroxyl groups), 931–928 cm^−1^ (ν_a_ AGU), 861–850 cm^−1^ (δ_oop_ C_1_–H), 765–758 cm^−1^ (ν_s_ AGU).

#### 2.4.6. Wide Angle X-ray Diffraction (WAXS)

The diffraction patterns and the crystalline fraction (F_C_) of samples were obtained with a Bruker model D8 Advance diffractometer (Bruker Corporation, Karlsruhe, Germany) equipped with a Goebel mirror and a Vantec PSD detector, using KαCu radiation filtered through Ni (λ = 1.54 Ǻ). The dried powder samples of the different modified starches were analyzed in an angular range 2θ recorded from 5 to 35°, with amplitude per step of 0.02°. The estimated error of 2θ was ± 0.2°. The sample holder used was 0.2 mm thick aluminum. The F_C_ of the different samples was calculated by Equation (5).

(5) $$F_{C}(\%)=\frac{A_{C}}{A_{C}{+A}_{a}}\times 100$$

where A_C_ is the area of the crystalline peaks and A_a_ is the area of the amorphous halo. For which the area corresponding to the crystalline zone and the total area under the curve (crystalline and amorphous area) was determined, following the iterative process described by Brückner [15].

#### 2.4.7. Thermogravimetric Analysis (TGA)

The thermal stability of all materials was determined through TGA using a TGA TAQ500 thermobalance (TA instruments, New Castle, DE, USA). The thermal decomposition of the samples was carried out in air atmosphere with a flow of 20 cm^3^/min. About 4–12 mg of the samples was weighed and heated from 50 to 800 °C at a rate of 10 °C/min. The integral method of Broido [16] allowed us to calculate the apparent activation energy, E_a_, for the decomposition of the different materials using Equation (6).

(6) $$\ln(\ln\left(\frac{1}{Y}\right))\;=-\frac{E_{a}}{R}\left(\frac{1}{T}\right)+\;\mathrm{constant}$$

where E_a_ is the activation energy in J/mol, T is the temperature in K, R is the universal constant of gases (J/mol K) and Y is the mass fraction of the sample not converted yet, which is calculated with the Equation (7).

(7) $$Y=\frac{W_{t}-W_{\infty }}{W_{0}-W_{\infty }}$$

where W_t_ is the sample mass at temperature T, W_0_ is the initial mass of the sample and W_∞_ is the mass of ash recorded at the maximum temperature of the analysis. All the amounts are expressed in mg.

#### 2.4.8. Scanning Electron Microscopy (SEM)

SEM characterization of starches was performed using Phillips XL30 equipment (SEMTech solutions, Massachusetts, USA) working at 25 kV and Au/Pd (80/20) coated samples. Particle size distributions were determined from the SEM images using the Fiji distribution in the ImageJ software (ImageJ for Microscopy, National Institutes of Health, Maryland, USA).

### 2.5. Water Adsorption Behavior in Different Media 

The swelling behavior in water, as well as the response to pH and ionic strength of the graft copolymers, was determined gravimetrically. For this, 50 mg of the sample was agitated for 24 h with 30 mL of distilled water, buffers solutions and in dissolved salts at different concentrations. After this time, the supernatant was removed, and the swollen hydrogel was weighed. The swelling of each sample was calculated by Equation (8).

(8) $$H^{\infty }\;(\%)=\frac{W_{t^{\infty }}-W_{0}}{W_{0}}\times 100$$

where H^∞^ is the percentage of hydrogel swelling at the equilibrium, W_t_ is the mass of hydrogel in mg at the equilibrium and W_0_ is the initial mass of the sample in mg previous to immersion. 

### 2.6. Controlled Release of Fertilizers

#### 2.6.1. Fertilizer Absorption (FL)

The superabsorbent starches (500 mg) were immersed in a concentrated solution of the fertilizer (0.5–10 g/L) for 24 h. Subsequently, the materials were filtered and dried at 40 °C for 3 days, the percentage of fertilizer absorbed was determined by gravimetry using Equation (9).

(9) $$\mathrm{FL}(\%)=\frac{W_{1}-W_{0}}{W_{0}}\times 100$$

where W_1_ and W_0_ are the masses of the hydrogel in mg after and before loading the fertilizer, respectively [17].

#### 2.6.2. Controlled Release of Fertilizers (FR)

**Scheme 1:**
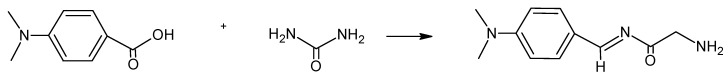
Ehrlich reaction.

For the release of urea, 100 mg of the loaded sample was introduced in 100 mL of distilled water, the release of the active species was allowed for specific times. After each period, 3 mL of the release medium was removed, refilling them with fresh distilled water [8,18]. The determination of the released urea content was made by spectrophotometry, using a UV-visible Techno Scientific model Genesys10S, following the methodology described by Watt and Chrisp [19], by forming a colored complex between urea and Ehrlich’s reagent, as shown in Scheme 1:

For the release of KNO_3_ and NH_4_NO_3,_ the same procedure used in the release of urea was followed except that the amount of released KNO_3_ or NH_4_NO_3_ was measured directly in the delivery medium by conductimetry (HM digital). For all fertilizers, the results were reported as percentage of fertilizer released, obtained through Equation (10) [20].

(10) $$\mathrm{FR}\left(\%\right)=\frac{M_{t}}{M_{T}}\times 100$$

where M_t_ and M_T_ are the masses of released fertilizer at each time and the total mass of the loaded fertilizer in the hydrogel, respectively, both expressed in mg.

## 3. Results

### 3.1. Characterization of Graft Copolymers

#### 3.1.1. Percentage of Insoluble Fractions I (%) and Content of –C(O)H and –COOH Groups

**Figure 1:**
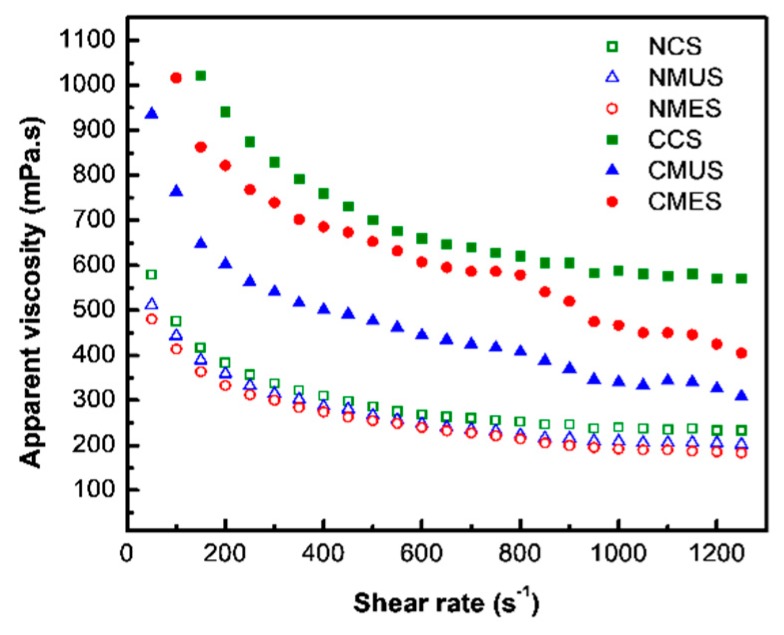
Apparent viscosity as a function of shear rate for the different samples.

The contents of –C(O)H and –COOH of the native starches and the graft copolymers based on corn starch (CCS), bitter cassava (CMES) and sweet cassava (CMUS) with IA are shown in Table 1. In general, there is a decrease in the content of –C(O)H while an increase in the content of –COOH groups with respect to the native starches is observed that indicates that both the oxidation of the –C(O)H to –COOH groups as the grafting of IA on the starch take place. The level of –COOH found means that the grafting reaction prevailed over the oxidation of –C(O)H to –COOH. In turn, high values of I (%) are obtained, which indicates the crosslinking of the materials resulting from the inclusion of –COOH groups in the polymer network. These results are confirmed by the increase in the apparent viscosity of the graft copolymer products due to the crosslinking of the polymer chains as depicted in Figure 1. The reinforcement that the crosslinking of the polymer chains imparts to the graft copolymer makes it more resistant to shear stress. Thus, the graft copolymers are more viscous with respect to their native starches and the order of apparent viscosity is in good agreement with its content of carboxyl groups.

#### 3.1.2. Wide Angle X-ray Diffraction (WAXS)

**Figure 2:**
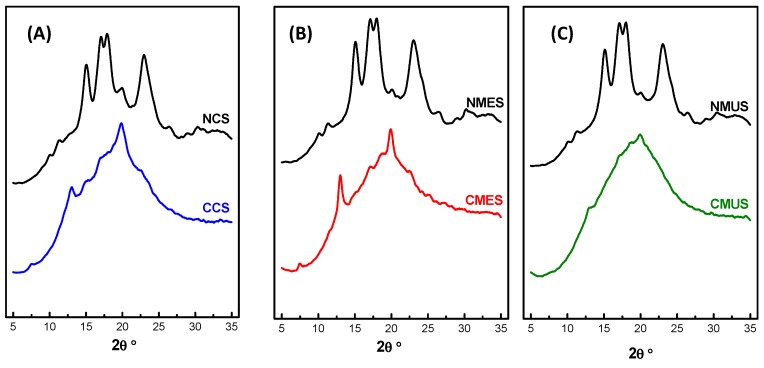
XRD profiles of (**A**) corn starch (CCS) grafted starch and its corresponding native corn starch (NCS), (**B**) bitter cassava (CMES) grafted starch and its corresponding native bitter cassava starch(NMES), and (**C**) sweet cassava (CMUS) grafted starch and its corresponding native sweet cassava starch (NMUS).

The diffractograms of the graft copolymers with their respective native starches are shown in Figure 2. In the NCS pattern (Figure 2A) two strong diffraction peaks were found overlapping at 2θ = 17.06° and 17.90°, and intermediate intensity diffraction peaks centered at 2θ = 15.05° and 22.96°, in addition to weak diffraction peaks at 10.05°, 11.35°, 19.95°, 26.40°, and 30.30° typical of crystalline structures A-type, corresponding to cereals starches [21,22,23].

The diffraction patterns of NMES (Figure 2B) and NMUS (Figure 2C) showed important similarities, with the overlapping of two strong diffraction peaks located at 2θ = 17.12° and 17.97°, two intermediate intensity peaks centered at 2θ = 15.12° and 23.03°, and weak diffraction peaks at 10.13°, 11.34°, 20.08°, 26.47°, 30.36°, and 32.86 °. Additionally, a low intensity peak found at 2θ = 5.51° in both materials demonstrates the presence of B-type crystals, characteristic of starches from tubers [21]. The crystalline structure of the studied native cassava starches corresponds to mixtures of polymorphisms A and B, according to previous research [24,25].

The graft copolymers presented three diffraction peaks characteristic of V-type crystals, which are centered at 2θ = 7.5°, 13°, and approximately 20°. In native starches, V-type crystals are in their amorphous state, with the graft copolymerization reaction, these crystals are rearranged and form crystalline lamellae [26]. In general, diffractograms of graft copolymers, with the exception of the mentioned peaks, show an amorphous halo, indicating that the copolymerization reaction had a great effect on the crystalline structure of the granule.

In the CCS WAXS pattern, compared to its native, it was observed that the overlapping diffraction peaks disappear and, in contrast, a shoulder centered between 2θ = 16.95–18.32° appear, the 15° peak is shown as a plateau centered between 2θ = 14.94–15.82° and finally the average intensity peak at 2θ = 22.96° is reduced to one of very low intensity centered at 2θ = 22.55°.

In the diffractograms of the cassava starches copolymers, CMES, and CMUS, the loss of the main diffraction peaks is evident compared to their native starches. CMES showed two very low intensity peaks centered at 2θ = 17.13° and 22.46°, as well as a small plateau centered between 2θ = 18.61–19.20°. In CMUS it is also appreciated that the strong diffraction peaks are transformed into a shoulder centered between 2θ = 17.10–18.54°, while the rest of the peaks disappeared.

The F_C_ of the graft copolymers is 6.01% for CCS, 8.23% for CMES and 2.58% for CMUS, which are far from the crystallinity of their starting starches, NCS = 29.61%, NMES = 38.40% and NMUS = 37.10%. When the copolymerization reaction takes place, the substitution in the –OH groups of the starch chain by bulky pendant chains of poly (itaconic acid), (PIA), occurs, which restricts the formation of starch-starch hydrogen bonds and consequently their crystallinity is reduced [9,27]. Therefore, this fact indicates that the copolymerization process affects the crystalline regions of the starch granule and that both amorphous and crystalline areas are involved in the chemical modification [28,29,30,31].

#### 3.1.3. Thermogravimetric Analysis

**Figure 3:**
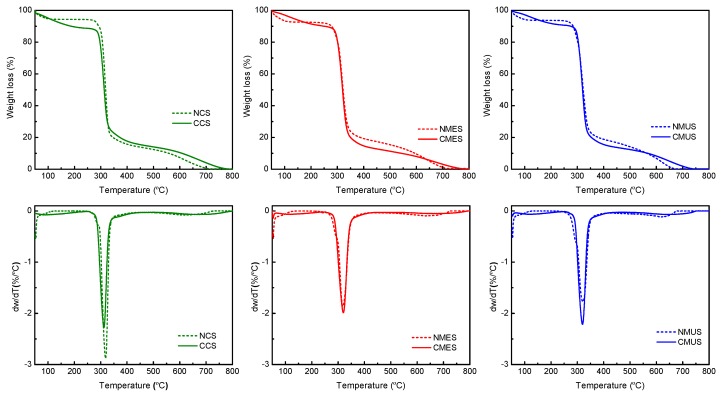
Thermograms of the different graft copolymers and their corresponding native starches.

TGA/DTG curves corresponding to the graft copolymers CCS, CMES, and CMUS are presented together with those of their respective native starches in Figure 3. In general, the thermal decomposition of the materials consisted of three stages, the details of which are shown in Table S1 of Supporting Information (SI). 

The initial stage of evaporation or loss of moisture is in the range of 50–235 °C, with a percentage of mass loss between 9–11%. Grafted samples exhibit higher values for both parameters compared to their native starches. This is explained by (1) the inclusion in the starch structure of the grafted chains of the PIA, which has functional groups of hydrophilic character, (2) the dehydration of the PIA chains, which leads to formation of polyanhydrides [32,33].

The main decomposition stage of the graft copolymers begins at temperatures greater than that of their corresponding starting starches. The greatest differences are observed for CCS and CMUS, with a delay in the decomposition of up to 21 °C. In the DTG of the native starches, a peak/shoulder is observed prior to the main decomposition, corresponding to the decarboxylation process [9]. However, this is not differentiable in the copolymers. The decrease in the number of differentiable stages in a derivative thermal degradation (DTG) curve does not indicate a decrease in the processes that are carried out since these can overlap [34], so the process of decarboxylation of the copolymer probably is carried out in conjunction with the main decomposition of the material. At the end of the main decomposition peak, which constituted the greatest mass loss for all materials, a shoulder was observed, which can be attributed to the decomposition of the grafted PIA [28,32,35]. In this sense, the inclusion of PIA chains in the structure of starch modified the process of decomposition of the material, with the addition of a new phase.

The third stage, corresponding to the complete decomposition and carbonization of intermediates formed during the previous phase [36], moved to higher temperatures in the copolymers, in comparison with their native starch, as shown in the DTG. This confirms a change in the thermal degradation process of the material, indicating the generation of more thermally stable species during the main decomposition.

The percentage of ash at 800 °C for the copolymers was lower than that of their corresponding starting starches, being as low as 0.021% for CCS. This decrease can be caused by (1) the existence in the structure of species with lower thermal stability that cause greater mass loss during the decomposition stages, as is the case of the PIA chains, (2) the increase in hydrophilicity of the material that increases the moisture content retained, thus, for the same sample mass, there is a greater percentage of mass loss in the dehydration stage.

The thermal stability of the copolymers was evaluated in terms of the initial temperature, the maximum temperature (T_max_), the E_a_ and the mass loss percentages in the different stages. The initial temperature of all the copolymers was higher than that of their corresponding natives, proving to be more thermally stable. For CMES, the T_max_, E_a_ and the percentage of mass loss of this stage increased with respect to its native, showing that the displacement of the T_max_ towards higher values is accompanied by a decrease in the degradation rate due to the inclusion of new processes such as decarboxylation of grafted PIA chains, as well as the possible excision of them from the starch skeleton. The same case was observed for CMUS, with the exception of T_max_, which did not suffer variation. On the other hand, a contrary behavior was obtained for CCS with a decrease in T_max_, E_a_ and the percentage of mass loss, demonstrating the breakdown of the main chain is promoting. However, this did not compromise the decomposition of the grafted PIA chains, in which case, the excision of them would initially be favored.

#### 3.1.4. Scanning Electron Microscopy

**Figure 4:**
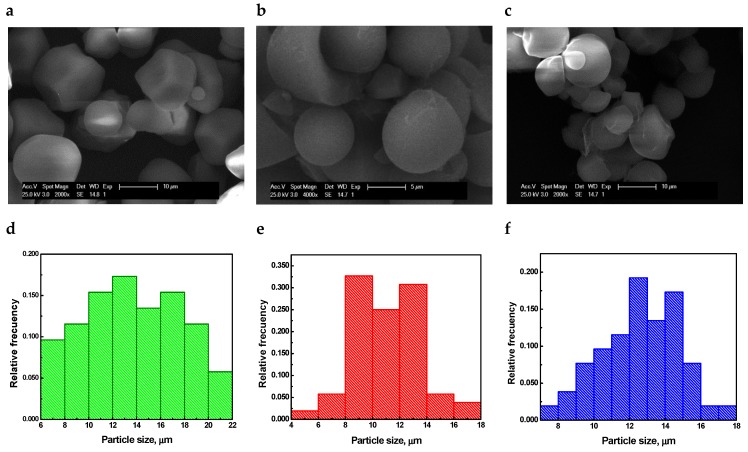
SEM images and the particle size distributions of (**a**,**d**) NCS, (**b**,**e**) NMES, and (**c**,**f**) NMUS, respectively.

In Figure 4, the micrographs corresponding to the NCS, NMES, and NMUS starches are shown. For NCS, dispersed and defined granules with irregular geometry, variable size, and completely smooth surface, with the presence on occasions of certain relief, were observed. A greater proportion of granules of polyhedral form were found, accompanied by a small population of granules of smaller size and circular shape. The characteristics observed in NCS have been previously described for native corn starches [9,36,37,38].

Regarding NMES and NMUS, both materials presented granules of variable shape and size, mostly ovals and truncated spheres, which are unique among their classification [25,37]. On the other hand, the surface is completely smooth for most of the granules, being in the rest a slight erosion, which can be explained by (1) the mechanical damage suffered by the granules in the extraction process and (2) the susceptibility to the spontaneous oxidation of these starches, which was evidenced by their content of –C(O)H and –COOH.

The granule size distributions for the three native starches presented a bimodal form. For NCS, the small granules had sizes between 6–9 µm and average particle diameter (PSA) of ca. 8 µm, whereas the medium granules were 10–22 µm in size with a PSA of 15 µm. In cassava starches, the small granules were between 6–10 µm with PSA of 9 µm for NMES and 8–10 µm with PSA of 9 µm for NMUS. The medium granules in NMES were found in a size range of 10–17 µm and PSA of 13 µm and the same for NMUS.

**Figure 5:**
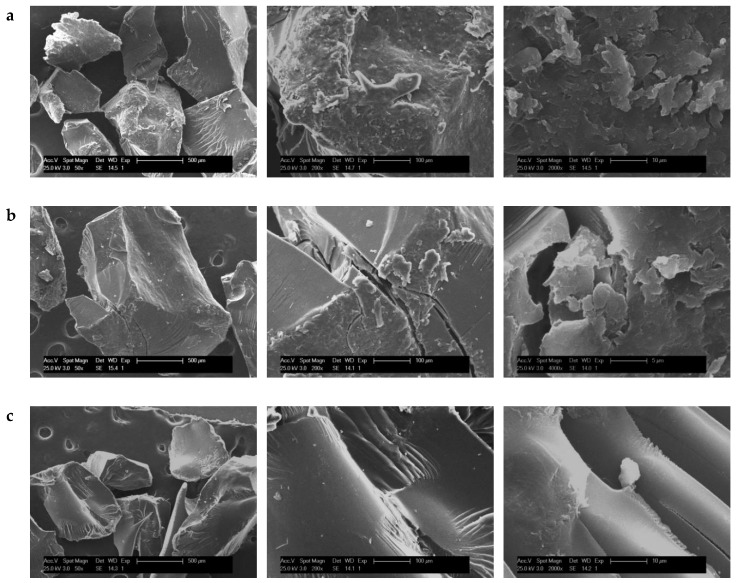
SEM images of (**a**) CCS, (**b**) CMES, and (**c**) CMUS at different augmentations.

The micrographs corresponding to graft copolymers are presented in Figure 5. For these materials, clear morphological differences are observed with respect to their corresponding natives due to the absence of either oval or spherical particles for CMES and CMUS, as well as polyhedral for CCS. The modification gives raise the development of particles of much larger size with sharp and defined edges, being between the values of 800–1200 µm. 

Structural heterogeneity is shown, with both porous and smooth areas, observing a greater proportion of the latter. There are also a large number of ridges on the surface, with varied ordering, parallel to radial. Such structural changes confirm the grafting of IA, forming complex structures where the starch and grafted chains of the PIA are entangled [38]. It is also evident the existence of compact porous surfaces for CCS and CMES, formed by the aggregation of granules [9,39], as well as cracks of different depths. Specifically, for CMUS, there are mostly smooth surfaces with the presence of channels and grooves. The pores, cracks, and channels observed in the copolymers form accessible regions for the interaction of the hydrophilic groups of the material with water and external stimuli [9,36].

### 3.2. Swelling Behavior in Different Media

The H^∞^ values for graft copolymers are ca. 1850 for CCS, 5550 for CMES, and 10,000 for CMUS. It was shown that in the 24 h after immersion in distilled water, the materials reached their H^∞^ and that these were greater than 100%, so they can be considered SASs. The largest H^∞^ values correspond to CMUS and CMES. The water absorption capacity of CMUS and CMES is accompanied by low crosslinking density and low crystalline fraction. CCS has the highest COOH content and the lowest H^∞^. This may be a consequence of the increase in the number of polymer-polymer interactions resulting from an excess of –COOH groups, responsible for the chain crosslinking through the formation of hydrogen bonds. Then there is an increase in the stiffness of the polymer network, limiting its swelling [6,9].

The behavior of SASs to ionic strength was evaluated by determining the H∞ with different salts (NaCl, KCl, NH_4_Cl, MgCl_2_, FeCl_3_) at different concentrations (0.1, 0.3, 0.5, 0.7, 0.9% w/v, as shown in Figures S1–S3 of SI). Swelling depends on the content of –COOH and ionic strength of the medium. The addition of salts to the medium leads to the contraction of the network because the cations of the dissociated salt shield the charges of the –COO^−^ in the polymeric network, reducing the electrostatic repulsions. In addition, the difference in osmotic pressure between the interior of the SAS and the external environment decreases, thus, all SASs exhibited a polyelectrolytic behavior [13]. From Figures S1–S3 of SI it is also observed that H^∞^ decreases with the increase in cation loads, so that monovalent cations > divalent cations > trivalent cations. This is because Mg^2+^ and Fe^3+^ form complexes with the –COO^−^ groups present in the copolymer. According to Liang et al. [40] this ionic crosslinking occurs mainly on the material surface.

Relative to monovalent ions, the value of H∞ was a function of the ionic radius of the cation, at a lower ionic radius greater H^∞^. Therefore, the order of H∞ in saline solutions was found to be: Na^+^ (0.95 Å) > K^+^ (1.33 Å) ≈ NH_4_^+^ (1.42 Å).

**Figure 6:**
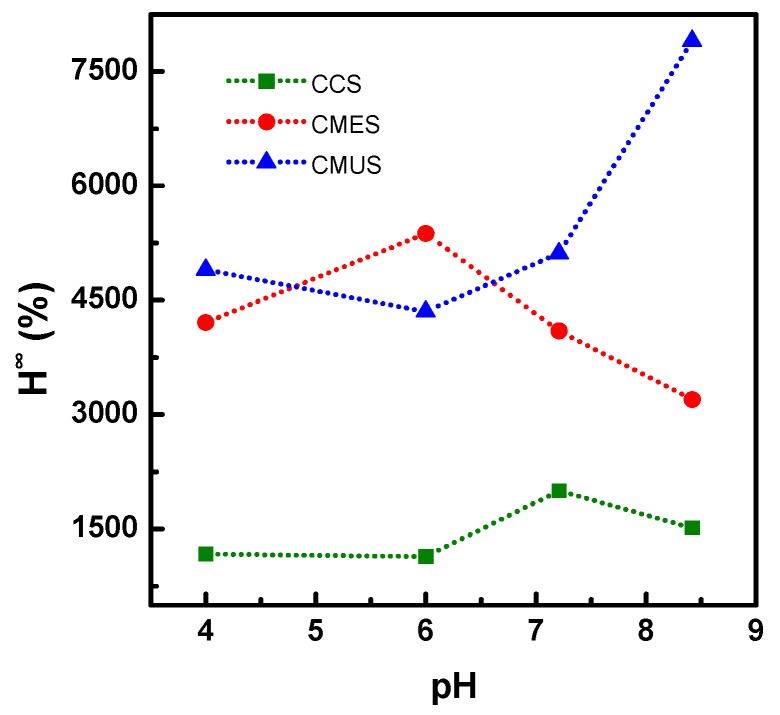
Swelling at the equilibrium of grafted copolymers as a function of pH.

The swelling capacity also depends on the pH. All copolymers showed responsiveness to pH changes (Figure 6), unlike their native starches due to the low content of –COOH groups. In the case of SASs, when the pH of the immersion medium is low, the degree of swelling is low because the functional groups are protonated (-COOH). In contrast, when the pH level increases above the pK_a_ of itaconic acid (pK_a1_ = 3.85 and pK_a2_ = 5.45) the number of fixed charges in the network also increases and generates anionic units (–COO^−^) that induce coulombic repulsions in the molecular chains, which lead to a greater expansion of the network and an increase in the percentage of swelling of the materials [41]. 

For CCS, the H∞ at pH = 4.00 and pH = 6.00 were similar and the lowest values in the pH range studied, demonstrating that in these conditions there was a greater polymer-polymer attraction, promoted by the –COOH groups. The drastic increase in H∞ with the increase in pH to 7.21 was the result of the ionization of the –COOH groups, which generates the electrostatic repulsion between the chains of the polymeric network and as a consequence, the existence of greater space for water entrance. The increase to pH = 8.4 caused a decrease in H∞ for CCS. This can be explained by 1) the presence in the medium of PO_4_^3−^ ions, which may cause a greater decrease in H∞ than Cl^−^ ions [8], and 2) the screening of the anionic groups due to the increase in the concentration of the mobile ions in solution [42], adding high content of –COOH groups, which at pH = 8.4 are completely ionized, causing a significant increase in the charge density of the material and limiting the access of highly polar molecules such as water [42].

The expulsion of water from CMES at pH above 6 is due to the fact that ionization begins at approximately the apparent pK_a_ value of IA. If the ionization of the group is complete, the swelling process stops. Far from increasing the pH, it increases the ionic strength, decreasing the osmotic pressure and causing the SASs to collapse [41]. In the case of CMUS, the swelling was not finished in the analyzed pH range, which indicates a lower degree of crosslinking as the insoluble fraction and crystalline fraction of this material was lower than for CMES. At high pH, –COOH groups were hidden in the network and not analyzed by the determination test thereof, which may be ionized causing a greater expansion of the network.

### 3.3. Controlled Fertilizer Release

#### 3.3.1. Fertilizer Absorption

**Figure 7:**
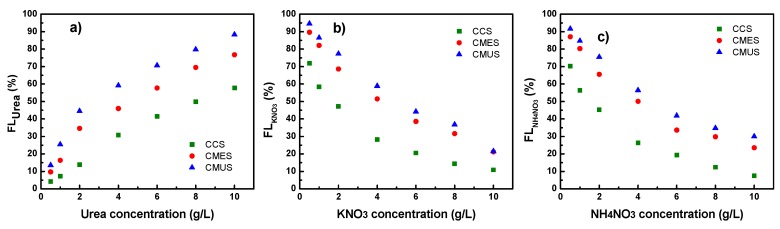
Fraction of loading in grafted starches: (**a**) urea, (**b**) KNO_3_, and (**c**) NH_4_NO_3_.

The urea loading was carried out at different urea concentrations (0.5–10 g/L) for 24 h at room temperature. Figure 7a shows the urea fraction of loading (FL_urea_) for grafted starches. The number of cracks and pronounced channels, observed by SEM for CMUS and CMES, favored the absorption of fertilizer.

FL_urea_ also increased with the decrease on I (%), the F_C_ and the increase in H∞. CMUS with the lowest I (%), the lowest FC and highest H∞ presented the highest FL, while CCS showed the lowest, due to its low H∞ and high value of I (%). The high content of -COOH groups in CCS prevented urea from entering, because of these groups promote polymer crosslinking.

The urea loading was strongly affected by the urea concentration. This may be due to the fact that fertilizer absorption occurs when the polymer matrix swells due to the ionization of the –COOH groups, which generates an electrostatic repulsion that allows the relaxation of the chains, thus increasing the entry of water molecules and the active species into the network [6,7]. Since urea is a neutral molecule, its loading is not affected by the repulsion forces of –COO^−^ in the polymer chains. Urea also has hydrophilic sites (–NH_2_) that improve the interactions between water and the polymeric network [39], and the urea absorption would be favored.

The absorption of KNO_3_ was also carried out at various concentrations of (0.5–10 g/L) for 24 h at room temperature and Figure 7b shows the FL_KNO3_ of starches. The following descending order was obtained CMUS > CMES > CCS at all concentrations. The higher FL_KNO3_ of CMES and CMUS can be attributed to its low crosslinking density and high H∞. For its part, CCS has a higher I (%) than CMES and CMUS, which resulted in lower absorption of agrochemicals. KNO_3_ is a strong electrolyte, which had a great influence on the H∞ of SASs, and therefore, on the charge of this fertilizer. 

On the other hand, the addition of NH_4_NO_3_ favors the combined nutrition of NH_4_^+^ and NO_3_^−^ for the benefit of plants. These forms of nitrogen are really available to plants, they also represent about 70% of total cations and anions. The absorption of NH_4_NO_3_ was similarly carried out at different concentrations for 24 h at room temperature and the results are shown in Figure 7c. The SASs were able to absorb more NH_4_NO_3_ at lower concentrations due to the action exerted by the ions dissolved in the medium. Even when the process was dominated by the chemisorption of NH_4_^+^ cations, NO_3_^−^ physisorption also occurs during loading (these results were confirmed by UV-vis spectroscopy by decreasing the NO_3_^−^ concentration in the charged medium depending on time, data not shown). Thus, the NH_4_^+^ cations interact electrostatically with the –COO^−^ to form -COO^−^NH_4_^+^ groups, which facilitates the adsorption of the NO_3_^−^ anions into the polymer network.

#### 3.3.2. Fertilizer Release

**Figure 8:**
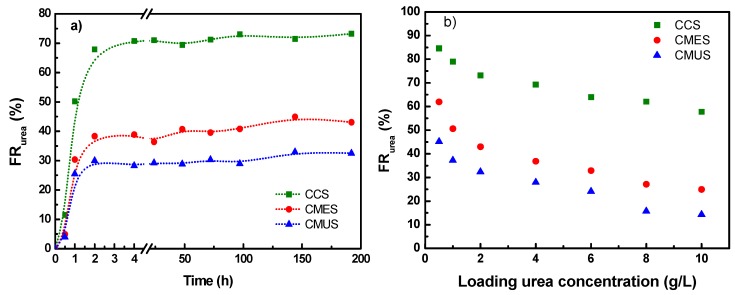
(**a**) Kinetics of urea release, and (**b**) urea release at different loading concentrations on grafted starches.

Figure 8a shows the urea release kinetics with an initial urea loading concentration of 2 g/L of the different SASs. These exhibited first order release behavior and showed a slightly increase in FR_urea_ after a time of approximately 100 h.

FR_urea_ decreases as the initial amount charged increases (see Figure 8b). This behavior can be related to (1) a process of desorption between the water and urea molecules due to the availability of a large free volume inside the starches, generated by the separation between the chains (low I (%)), which causes the reabsorption of the active species within the polymeric network [8], and (2) the decrease in the diameter of the pores or depth of the channels inside and at the surface of the material due to the urea accumulation into them (high FL), which hinders the entrance of water into the matrix [7]. Both phenomena favor the reduction in the amount of fertilizer released.

**Figure 9:**
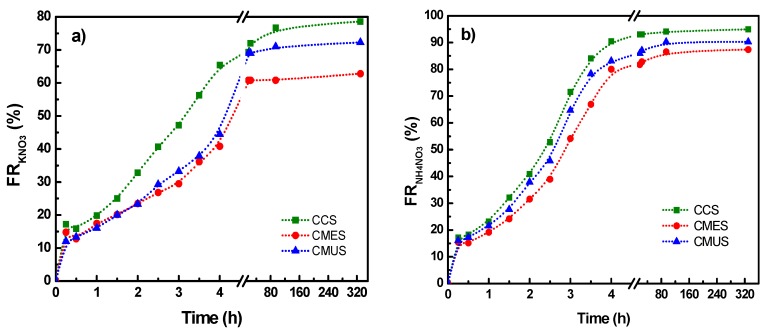
Release kinetics of (**a**) KNO_3_ with an initial loading concentration of 10 g/Land (**b**) NH_4_NO_3_ at with an initial loading concentration of 0.5 g/L on grafted starches.

**Figure 10:**
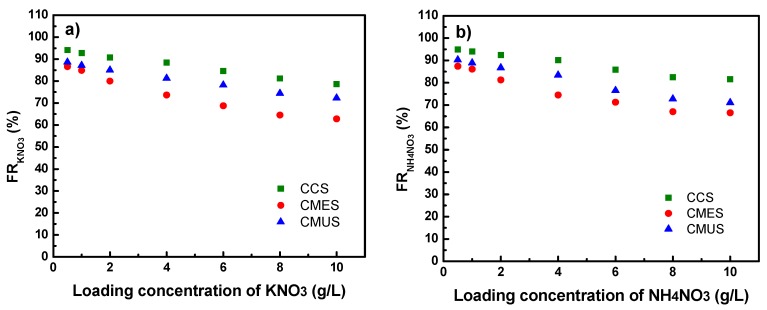
Fraction release of (**a**) KNO_3_ and (**b**) NH_4_NO_3_ at different loading concentrations.

Figure 9 shows the release kinetics of KNO_3_ and NH_4_NO_3_, respectively, of SASs. In both cases, the behavior observed in the curves that represent the release kinetics consists of three zones or stages, which give it a characteristic “S” shape. A first stage where the fertilizer is slowly released, followed by a rapid release zone where the greatest amount of fertilizer is released in all materials and finally, an area where the release reaches a constant value [18,43]. The rapid increase in KNO_3_ and NH_4_NO_3_ between t = 0 and t = 0.5 h is due to the occurrence of a burst effect due to the amount of fertilizer deposited on the surface of the charged hydrogel [8]. The increase in the release rate in the second zone is attributed to the progressive expansion of the polymer matrix due to its swelling [43]. This behavior is accentuated with the increase in the initial concentration of fertilizer loaded (see Figure 10).

CMUS was the grafted starch with the highest loading amount of fertilizer (KNO_3_ or NH_4_NO_3_) and the lowest degree of crosslinking, demonstrating the influence of these factors on the controlled release of ionic fertilizers. The high FR of CCS with respect to its low fertilizer load can be explained by the absorption of ionic fertilizer preferentially in surface areas of SAS starches. This is due to the high I (%) of this material, which makes impossible to enter either water, KNO_3_ or NH_4_NO_3_ [42]. The fertilizer deposited at the surface or more external areas of the network can be released more easily, that is why this material reached equilibrium faster than the rest.

## 4. Conclusions

Modification of corn, bitter yucca and sweet yucca starches by IA graft copolymerization was corroborated with the results obtained from the –C(O)H and –COOH content of groups, FTIR, TGA, XRD, and SEM. The materials turned out to be superabsorbent polymers capable of responding to external stimuli such as pH and ionic strength, both due to the characteristics granted by the inclusion of the –COOH groups to the starch structure. Likewise, the characteristics obtained by the starches of different botanical sources thanks to the modification, making them suitable materials to be used as polymeric matrices in the absorption and release of fertilizers. An increase of fertilizer concentration from 0.5 g/L to 10 g/L led to an increase in urea absorption and a decrease in KNO_3_ and NH_4_NO_3_ adsorption. In turn, the fertilizer release behavior is different, an increase in the concentration of loaded fertilizer led to a decrease in FR for the three fertilizers studied. Cassava SAS starch showed greater absorption capacity for all fertilizers, releasing NH_4_NO_3_ in greater amounts, retaining urea in greater proportion during the release in water.

## Figures and Tables

**Table 1:** I (%) and contents of –C(O)H and –COOH groups in the graft copolymers.

Sample	–C(O)H (%)	–COOH (%)	I (%)
NCS	0.1643 ± 0.0127	0.1510 ± 0.0804	79.5
NMES	ND	0.3144 ± 0.0001	85.4
NMUS	0.0224 ± 0.0001	0.2676 ± 0.0410	89.1
CCS	0.047 ± 0.063	0.441 ± 0.017	98.4
CMES	0.015 ± 0.037	0.258 ± 0.009	89.5
CMUS	0.046 ± 0.034	0.157 ± 0.009	87.7

ND: No detected. The results are the average of the triplicate determination and are expressed as mean ± standard deviation.

## Supplementary Materials

The following are available online at https://www.mdpi.com/1996-1944/12/21/3493/s1, Figure S1: Swelling equilibrium of grafted copolymer CCS as a function of salt concentration for different salts, Figure S2: Swelling equilibrium of grafted copolymer CMES as a function of salt concentration for different salts, Figure S3: Swelling equilibrium of grafted copolymer CMUS as a function of salt concentration for different salts, Table S1: Thermal parameters of native and grafted starch samples.
