# Supplementary material for: Environmentally Friendly Fertilizers Based on Starch Superabsorbents
Source: Materials (Basel). 2019 Oct 25;12(21):3493. doi: 10.3390/ma12213493 (PMC6862305; doi:10.3390/ma12213493)
Supplement: Supplementary file 1 [file materials-12-03493-s001.pdf]

materials

Supplementary

Environmentally Friendly Fertilizers Based on Starch Superabsorbents

Orietta León[1,*], Diana Soto [1], Jesús González [1], Carlos Piña [1], Alexandra Muñoz-Bonilla [2,3] and Marta Fernandez-García [2,3,*]

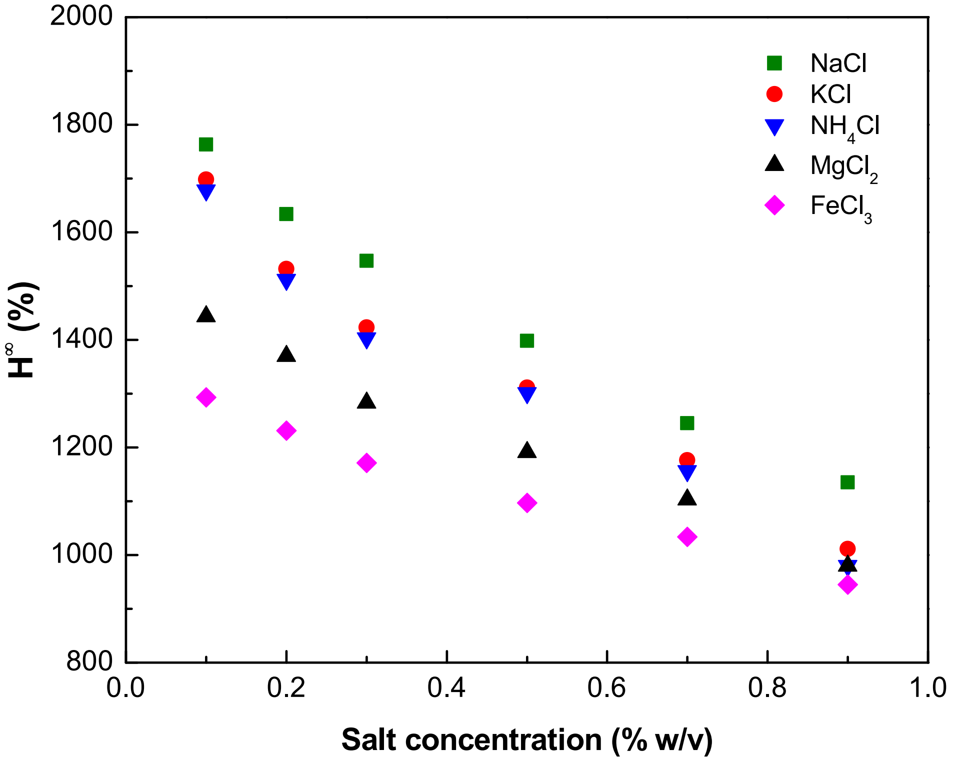

Figure S1. Swelling equilibrium of grafted copolymer CCS as a function of salt concentration for different salts.

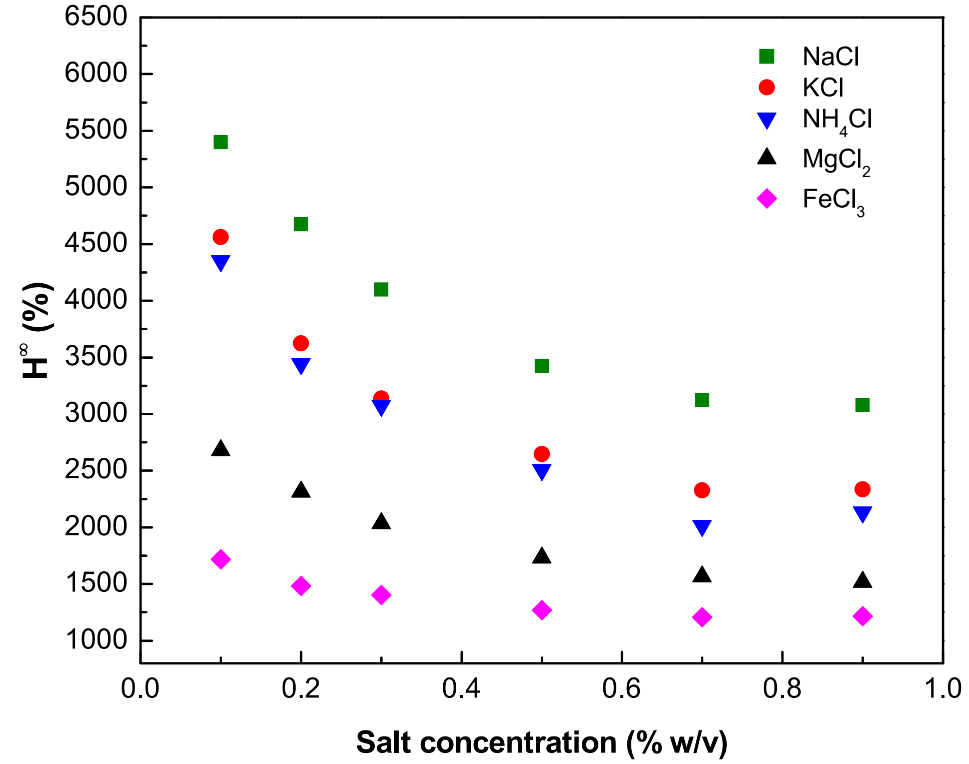

Figure S2. Swelling equilibrium of grafted copolymer CMES as a function of salt concentration for different salts.

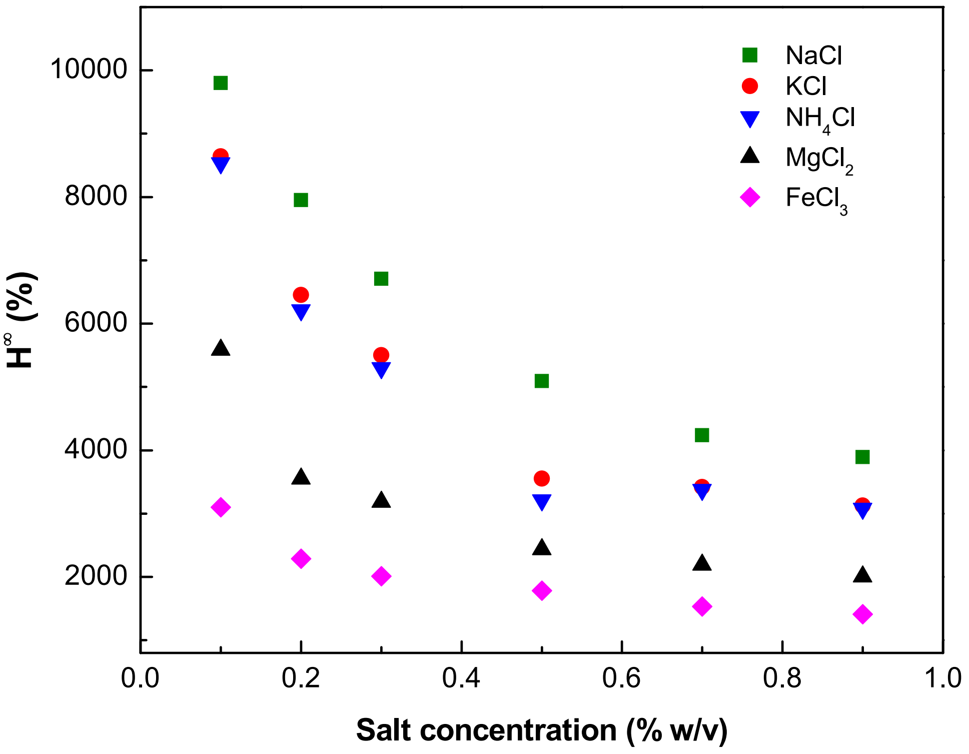

Figure S3. Swelling equilibrium of grafted copolymer CMUS as a function of salt concentration for different salts.

Table S1. Thermal parameters of native and grafted starch samples.

Sample	Step	Temperature (°C)	T_{max} (°C)	Weight loss (%)	E_a (KJ/mol)	Ash (%)
NCS	1. Evaporation	50–121		5.6	ND	0.1448
	2.1. Decarboxylation	245–292		4.2	89.29	
	2.2. Main decomposition	292–434	318	77.7	231.46	
	3. Carbonization	507–728		12.4	34.33	
NMES	1. Evaporation	50–121		7.2	ND	0.4184
	2.1. Decarboxylation	253–298		9.9	91.20	
	2.2. Main decomposition	298–389	318	68.3	145.50	
	3. Carbonization	546–725		14.1	28.97	
NMUS	1. Evaporation	50–120		6.2	ND	0.2329
	2.1. Decarboxylation	235–297		10.6	110.74	
	2.2. Main decomposition	297–411	320	69.1	145.08	
	3. Carbonization	514–675		13.9	46.72	
CCS	1. Evaporation	50–229		11.0	ND	0.0210
	2.1. Main decomposition	258–340	313	64.4	182.99	
	2.2. PIA decomposition	340–441		8.4	13.67	
	3. Carbonization	558–784		12.2	31.44	
CMES	1. Evaporation	50–230		70.9	ND	0.1681
	2.1. Main decomposition	254–356	320	5.6	162.70	
	2.2. PIA decomposition	356–428		9.0	17.80	
	3. Carbonization	569–800		9.1	29.68	
CMUS	1. Evaporation	50–235		70.6	ND	0.1386
	2.1. Main decomposition	256–353	320	5.7	182.91	
	2.2. PIA decomposition	353–427		11.2	15.19	
	3. Carbonization	569–800		70.9	54.01	

ND: No determined; T_{max}: Temperature at the maximum

© 2019 by the authors. Submitted for possible open access publication under the terms and conditions of the Creative Commons Attribution (CC BY) license (http://creativecommons.org/licenses/by/4.0/).
